# Catamenial Pneumothorax: A Rare Diagnosis Among Menstruating Women

**DOI:** 10.7759/cureus.45769

**Published:** 2023-09-22

**Authors:** Kimberly Nguyen, Brian G Nudelman, Jorge Quiros, Marianne Cortes, Cristina Savu

**Affiliations:** 1 Internal Medicine, Broward Health Medical Center, Fort Lauderdale, USA; 2 Internal Medicine, Memorial Healthcare, Pembroke Pines, USA; 3 Osteopathic Medicine, Nova Southeastern University Dr. Kiran C. Patel College of Osteopathic Medicine, Davie, USA

**Keywords:** hemopneumothorax, catamenial pneumothorax, spontaneous pneumothorax, spontaneous, menstrual, obgyn, endometriosis, pulmonology, pneumothorax, catamenial

## Abstract

Catamenial Pneumothorax is a rare condition often associated with endometriosis in menstruating women. Due to the rarity of this condition, its etiology is not well studied and, thus, effective treatment regimens have not been well established. We present a case of a 21-year-old female with no significant past medical history who developed recurrent episodes of spontaneous pneumothorax, chronologically associated with her menstrual cycle. This pattern is known as the *sine qua non *criteria and is one of the only established criteria in current literature for diagnosing catamenial pneumothorax. Our aim with this case report is to expand the current collection of published knowledge about this rare condition and to bring awareness so that those affected by catamenial pneumothorax can be diagnosed and treated more efficiently. Additional research on the pathophysiology of this disease needs to be done to aid in the development of effective treatment regimens.

## Introduction

Catamenial Pneumothorax (CP) is an extremely rare, but very dangerous condition. While it only comprises 3%-6% of all spontaneous pneumothorax cases among menstruating women, its unique presentation makes CP an important subject for research. The main criteria for the diagnosis of catamenial pneumothorax are known as the *sine qua non* criteria, which is spontaneous pneumothorax within 72 hours before or after the start of a menstrual period. In addition to this, CP often presents with concomitant endometriosis, right-sided location, and pleural lesions; however, these criteria do not have to be met for diagnosis. While the exact cause of CP has not been determined there are four main theories that explain its possible etiology. The Physiological Theory postulates that increased levels of prostaglandin F2 during menses constrict the bronchioles and blood vessels, leading to alveolar rupture and pneumothorax. Next, the Migrational Theory explains that endometrial cells may migrate from the uterus to the lesser pelvis, eventually causing damage to the diaphragm so they can pass into the thoracic cavity and invade the pleura. The third endometrial-based theory known as the Microembolic-Metastatic Theory says that endometrial cells can travel through the blood or lymph to the pleura where they can necrose and cause damage that leads to pneumothorax. Lastly, the Diaphragmatic Air Theory says that air passes from the fallopian tubes through small fenestrations in the diaphragm to the pleural cavity which eventually leads to CP. While plausible none of these theories fully explain the presentation of CP, underscoring the importance of adding new cases to the literature [1]. We are discussing a case of CP in a 21-year-old with recurrent right-sided pneumothorax during her menstrual periods who underwent surgical intervention and hormonal treatment.

## Case presentation

**Figure 1:**
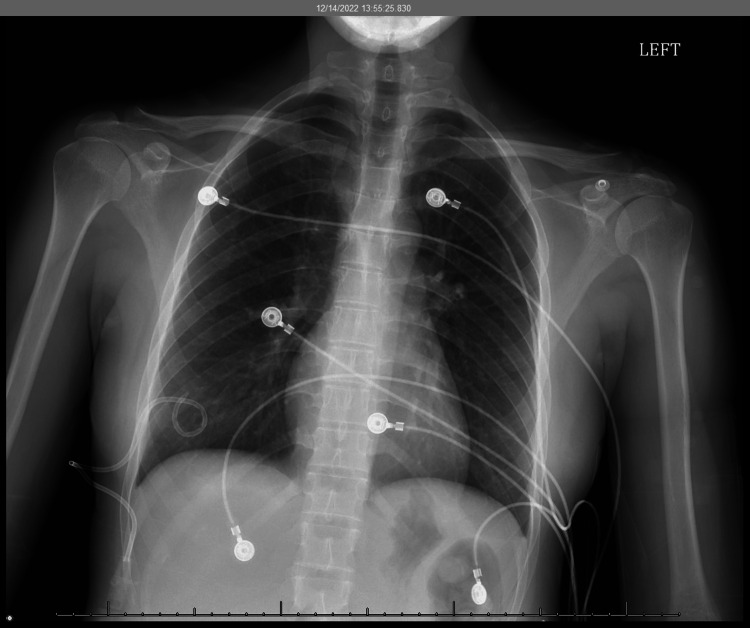
Initial chest x-ray Impression: Right pleural pigtail catheter tip in the right lung base. No pneumothorax and no effusions.

A 21-year-old G0P0 female presents to the ED as a transfer from another hospital for recurrent right-sided spontaneous pneumothorax (PTX). She had no significant past medical history, or past surgical history and denied any recent illness or symptoms. She is fully vaccinated for COVID-19 and denies any known positive results. She does not take oral contraceptives. She initially presented to another hospital with severe chest pain with shortness of breath which started two to three hours prior to her menstrual cycle. The chest pain was rated 6/10, described as sharp, sudden, and localized to the right. Her pain worsened with inspiration. She also reported an associated near-syncopal episode however syncope workup was negative. Her initial chest x-ray (CXR) revealed a 25% PTX on the right. She was treated with 10 L/min of oxygen via a non-rebreather and discharged home with a resolution of her symptoms which required no other intervention. Two weeks later, she presented again to the ER and was found to have a recurrent 20% right-sided PTX. The onset of her symptoms was within 48-72 hours of menses onset. During this second admission, her symptoms did not resolve with a non-rebreather mask. She was evaluated by the cardiothoracic team and a pigtail catheter was placed prior to the patient being transferred to our hospital for video-assisted thoracoscopic surgery. On arrival, the patient’s blood pressure was 128/88 mmHg, heart rate 98 bpm, respiratory rate of 22 cycles/min, and SpO2 of 97% on ambient air. Initial lab work was unremarkable aside from a mildly elevated WBC count of 11.0 and HgB of 11.5. She was immediately admitted to the intensive care unit for further monitoring and assessment. The patient underwent an initial CXR (Figure 1). The image findings confirmed the right pleural pigtail catheter tip in the right lung base. No pneumothorax and no effusions.

**Figure 2:**
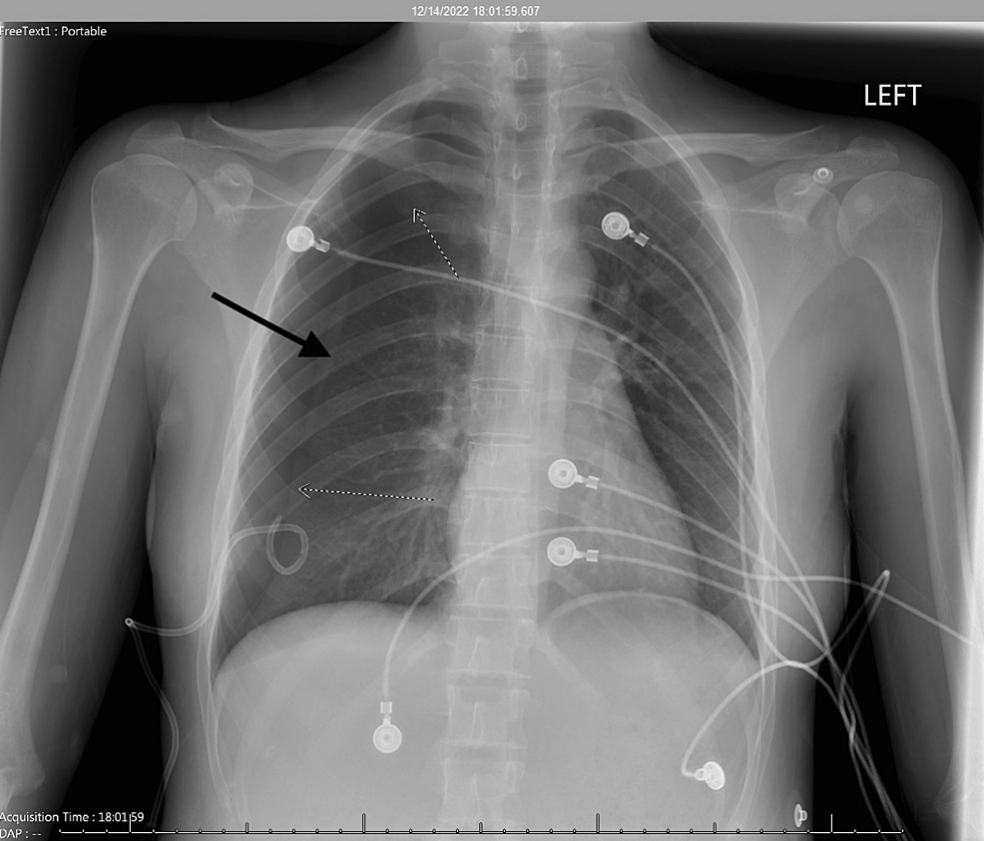
Chest x-ray #2 Impression: Pigtail catheter over the right lower lung. There has been interval development of a moderate to large right pneumothorax measuring approximately 50%, which has developed since the prior examination performed earlier the same day.  Mediastinal shift to the left. The lungs are otherwise clear. No left pneumothorax. No pleural effusion.

Shortly after arrival to the ICU, the patient began to complain of pain in the right side of the chest which she described as exactly as the pain she felt during her previous episode of pneumothorax. A CXR was done and confirmed a moderate to large pneumothorax (Figure 2).

**Figure 3:**
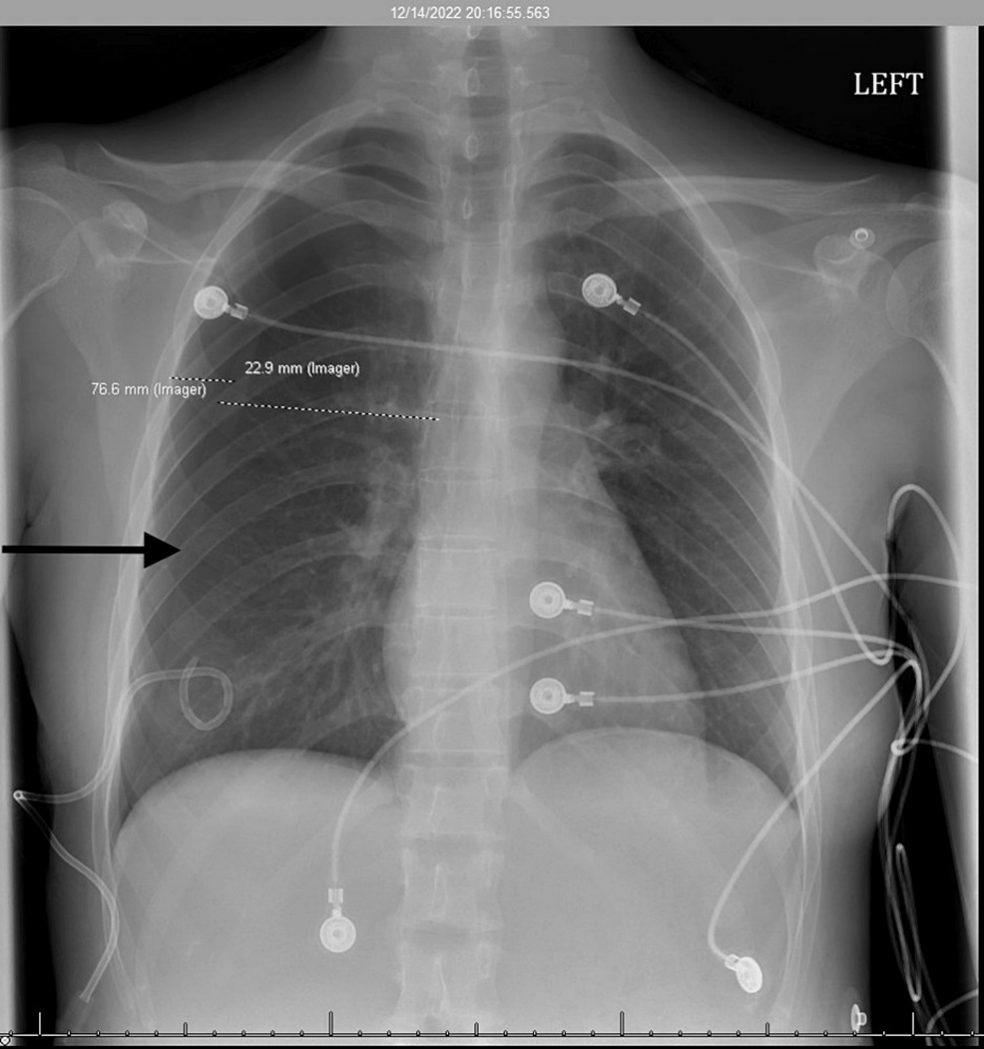
Chest x-ray #3 Impression: No consolidation, mass or edema. No pleural effusion. There is interval decrease in the size of the right pneumothorax measured approximately 40%.

The patient was placed on 2 2-liter nasal cannula. A pigtail catheter was assessed. There was no defect or obstruction to the catheter. There was no leak, and it was set to suction at -20cmH_2_O. The patient continued to complain of worsening chest pain and shortness of breath. A CXR was done and confirmed an interval decrease of the pneumothorax around 40% (Figure 3).

**Figure 4:**
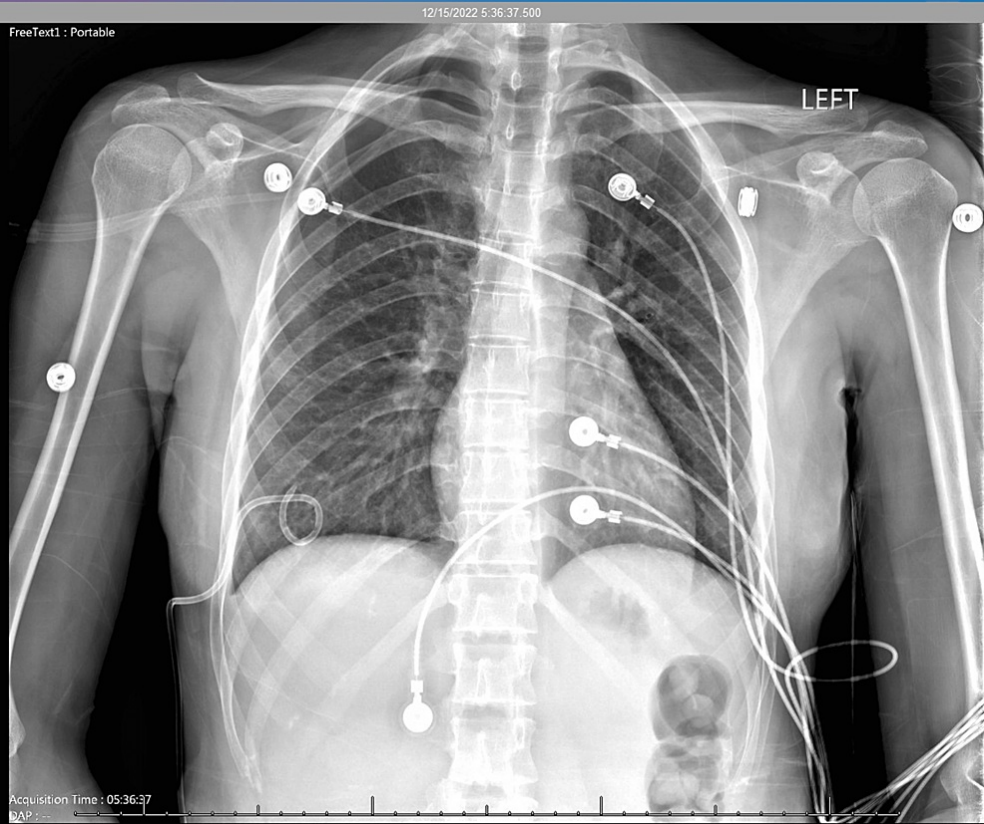
Chest x-ray #4 Impression: No new significant focal consolidation identified with continued interval improvement of right pneumothorax, now measuring approximately 15%-20%.

A CXR the following morning confirmed continued improvement of the pneumothorax (Figure 4).

**Figure 5:**
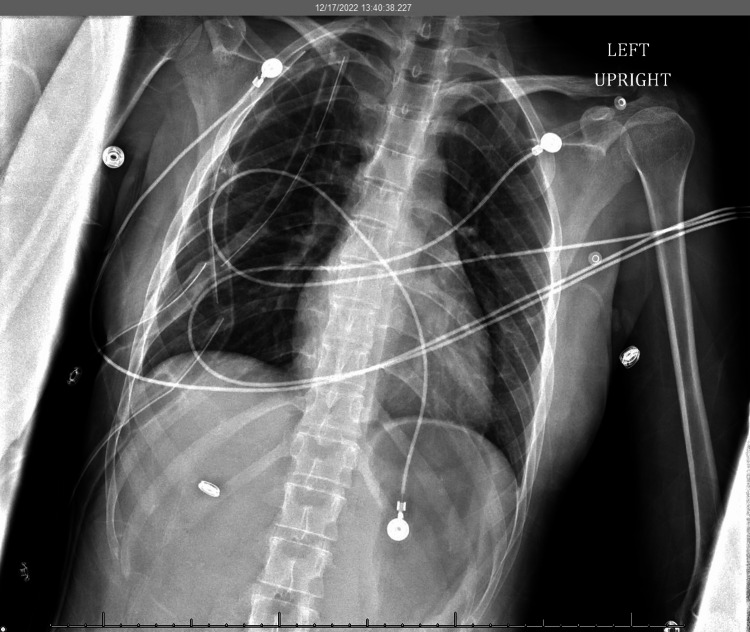
Chest x-ray done post VATS procedure Impression: Two large bore right-sided chest tubes placed. Lungs are well aerated and clear. There is no appreciable pneumothorax.

The patient was evaluated by the cardiothoracic surgical team and the team concluded her symptoms were most likely secondary to recurrent right pneumothorax due to endometriosis. The patient underwent right-sided video-assisted thoracoscopy, resection of the right upper lobe implant, resection of the right lower lobe implant, and mechanical pleurodesis. The hemithorax was found to have multiple suspected implants on the chest wall and diaphragm that were biopsied. There were also large implants identified on the right upper lobe and right lower lobe, which were removed and sent for pathology. Mechanical pleurodesis was performed and bupivacaine was injected at the intercostal spaces at each level. Chest tubes were placed and connected to suction and the lung was re-inflated. Post-operative CXR showed no appreciable pneumothorax (Figure 5).

**Figure 6:**
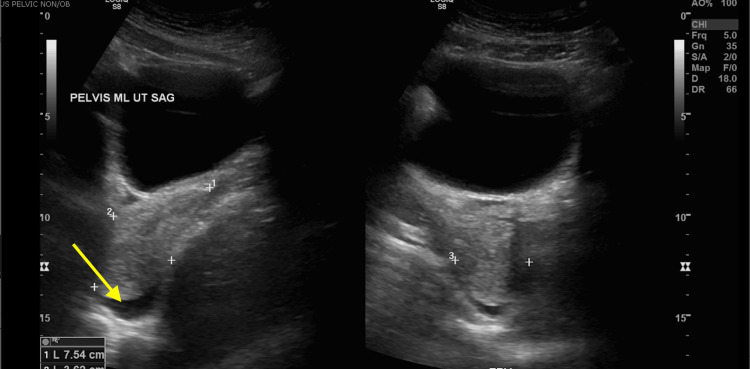
(Left, right) Pelvic ultrasound Impression: Uterus size of 7.5cm craniocaudal. Small volume pelvic free fluid. Otherwise negative study.

The following day she was evaluated by the obstetrics and gynecology team, who supported that her pneumothorax is likely secondary to Catamenial Pneumothorax associated with endometriosis. An ultrasound of the pelvis was ordered which confirmed a uterus size of 7.5cm craniocaudal and a small volume of pelvic free fluid (Figure 6). A long discussion was had with the patient and family about the causes, symptomatology, and treatment of this condition. She was given options of conservative management versus oral contraception versus gonadotropin use.

During post-operative days 3-5, the patient experienced continued resolution of her previous symptoms which were confirmed by daily CXRs which showed no appreciable pneumothorax and clear lungs bilaterally. Surgical pathology reports found lung parenchyma with congestion and hemorrhage as well as reactive changes in focal pleura with no evidence of abnormal infiltrates in the right upper lobe or lower lobes. In conclusion, the pathology report found soft tissue and pleural with chronic inflammation, granulation tissue, and reactive changes which were negative for endometriosis.

Prior to the patient's discharge home, we performed an extensive literature review in regard to the risks for recurrence. Through collaboration with the obstetrics and gynecology (OB/GYN) team, it was decided that the superior treatment option for the patient was hormonal management with a GnRH agonist. She was advised to complete three to six months of therapy. The patient was also told to consider possible laparoscopy in the future, but the team did not find any medical necessity or benefit at the time. The patient was discharged home post-operative day 5 with Aygestin 5 mg orally daily, Lupron Depot 11.25 mg/three months intramuscular powder for injection, and extended release 11.25 mg for 90 days. 

## Discussion

This presentation demonstrates a rare and interesting case of catamenial pneumothorax in a 21-year-old female with a past medical history of endometriosis. Our patient met the *sine qua non* criteria during both of her admissions. Additionally, her presentation with pneumothorax on the right side and a right lower lobe pleural implant further suggest the diagnosis of CP [1]. Literature suggests that the best course of action for the treatment of CP is surgical intervention, specifically video-assisted thoracoscopic surgery (VATS). In addition to resection of the right lower lobe specimen, cardiothoracic surgery performed a pleurodesis. One study found a slower rate of recurrence of CP in those who receive pleurodesis in addition to removal of all pleural and diaphragmatic foci which was 61 vs 24 months [2]. Additionally, the use of pleurodesis alone is not recommended as demonstrated in a study done by Leong et al. [3]. As per Ali and Surani, the indications for pleurodesis include recurrent pneumothorax, recurrent pleural effusion, and most often malignant pleural effusion. Contraindications include pleural elastance > or = 19 cmH_2_O on pleural manometry, previous thoracic radiation, and prolonged chest tube duration of 10 days or more [4]. Combining VATS, pleurodesis, mesh placement on diaphragmatic lesions and hormonal therapy has shown to be a more effective approach. Our patient had the removal of all suspected endometrial tissue in her thoracic cavity; however, there were no diaphragmatic lesions observed on the surgical report, and thus no mesh was placed. While our pathology results did not confirm the presence of endometrial cells, they did recommend clinical correlation for our diagnosis. Additionally, in a review by Marjanski et al., they explained that there are no pathology or radiology findings that give a definitive diagnosis of catamenial pneumothorax. Instead, the diagnosis is generally based on *sine qua non* and the clinical picture [1].

In a study by Attaran et al. on 12 patients with CP, it was found that the addition of hormonal treatment helped prevent the recurrence of PTX. As recommended by this study, our patient was started on hormonal therapy with Lupron and norethindrone and will be managed by a gynecological specialist [5]. Interestingly, another study by Subotic et al. showed no CP recurrence within six months of surgical intervention despite a lack of post-operative hormone treatment. The sample size of this study was significantly smaller with only four patients, so our team decided to continue with the more widely accepted treatment course [6].

One of the more interesting features in our case is the lack of diaphragmatic lesions that are often seen in many other cases of catamenial pneumothorax. We propose that this may be due to the Microembolic/Metastatic Theory of CP. In this theory, the endometrial tissue would not have to cross the diaphragm to reach the pleura because it would travel directly through the blood or lymphatic systems. A study from 1996 by Joseph and Sahn supports that the diagnosis of thoracic endometriosis and CP is generally a clinical diagnosis and not necessarily pathological [7]. We also attribute this more mild pathological presentation to be due to a very early stage of the disease. Our patient is younger than most women who present with CP as the mean age is generally 32-35 years old [1,8]. It is possible that with time she may start to develop lesions on her diaphragm, however, we aim to prevent this damage with the addition of hormonal therapy. Long-term follow-up every six months with OB/GYN and cardiothoracic surgery was also recommended to the patient. While hormonal therapy will only be used for six months due to the risk of significant side effects, we hope that the combination of VATS and pleurodesis will help prevent any future recurrences.

## Conclusions

In summary, our report demonstrates a case of catamenial pneumothorax in a patient with endometriosis. The occurrence of this condition is very rare and only comprises a very small amount of all spontaneous pneumothorax cases in young women. The documentation and analysis of each of these cases can help us to discover new etiologies and adjust the treatment course for all CP patients in the future. For now, VATS procedure, pleurodesis and diaphragmatic mesh placement along with adjunctive hormonal therapy has shown encouraging results in preventing recurrence of CP. We will continue to monitor our patient for any new episodes of pneumothorax and we encourage other physicians to consider catamenial pneumothorax if they are presented with a similar situation.
